# Differential Response of High-Elevation Planktonic Bacterial Community Structure and Metabolism to Experimental Nutrient Enrichment

**DOI:** 10.1371/journal.pone.0018320

**Published:** 2011-03-31

**Authors:** Craig E. Nelson, Craig A. Carlson

**Affiliations:** Marine Science Institute and Department of Ecology, Evolution and Marine Biology, University of California Santa Barbara, Santa Barbara, California, United States of America; Argonne National Laboratory, United States of America

## Abstract

Nutrient enrichment of high-elevation freshwater ecosystems by atmospheric deposition is increasing worldwide, and bacteria are a key conduit for the metabolism of organic matter in these oligotrophic environments. We conducted two distinct in situ microcosm experiments in a high-elevation lake (Emerald Lake, Sierra Nevada, California, USA) to evaluate responses in bacterioplankton growth, carbon utilization, and community structure to short-term enrichment by nitrate and phosphate. The first experiment, conducted just following ice-off, employed dark dilution culture to directly assess the impact of nutrients on bacterioplankton growth and consumption of terrigenous dissolved organic matter during snowmelt. The second experiment, conducted in transparent microcosms during autumn overturn, examined how bacterioplankton in unmanipulated microbial communities responded to nutrients concomitant with increasing phytoplankton-derived organic matter. In both experiments, phosphate enrichment (but not nitrate) caused significant increases in bacterioplankton growth, changed particulate organic stoichiometry, and induced shifts in bacterial community composition, including consistent declines in the relative abundance of Actinobacteria. The dark dilution culture showed a significant increase in dissolved organic carbon removal in response to phosphate enrichment. In transparent microcosms nutrient enrichment had no effect on concentrations of chlorophyll, carbon, or the fluorescence characteristics of dissolved organic matter, suggesting that bacterioplankton responses were independent of phytoplankton responses. These results demonstrate that bacterioplankton communities in unproductive high-elevation habitats can rapidly alter their taxonomic composition and metabolism in response to short-term phosphate enrichment. Our results reinforce the key role that phosphorus plays in oligotrophic lake ecosystems, clarify the nature of bacterioplankton nutrient limitation, and emphasize that evaluation of eutrophication in these habitats should incorporate heterotrophic microbial communities and processes.

## Introduction

Despite their relative isolation from human populations, high-elevation ecosystems are increasingly affected by global change. Warming trends are disproportionately altering these habitats [1], [2], and there is continuing evidence for large-scale anthropogenic depositional impacts to remote montane environments [3]–[5]. Atmospheric deposition of anthropogenic nitrogen has generated broad biogeochemical and ecological impacts in high-elevation regions of the western United States [6]. The interacting effects of acidification and climate on high-elevation environments throughout eastern North America and northern Europe in the latter half of the 20^th^ century are well-documented [7], [8]. In the Sierra Nevada of California (USA), atmospheric deposition of nitrogen (N) and phosphorus (P) represents a large fraction of the nutrient inputs to lakes at elevations greater than 2500 m above sea level (a.s.l) [9]. Two decades of monitoring suggest that lakes in the Sierra Nevada are undergoing eutrophication as well as a regional shift in nutrient limitation status [10], [11].

Evidence for eutrophication in these habitats is based largely on increases in phytoplankton abundance inferred from long-term trends in particulate organic matter (e.g. [11]). However, oligotrophic pelagic habitats such as high-elevation lakes are understood to support a predominantly microbial food web, where bacterioplankton and their grazers serve as a major link in the transfer of dissolved organic matter (DOM) to higher trophic levels [12], [13]. Investigating the effect of nutrient enrichment on bacterial community structure, growth, and consumption of DOM may therefore be highly relevant to understanding the eutrophication process in these highly dilute waters.

Studies simultaneously evaluating the direct impact of nutrient enrichment on bacterioplankton growth, DOM consumption, and community composition are rare, and are highly relevant to understanding how eutrophication may impact oligotrophic environments such as high-elevation lakes. Previous work investigating bacterioplankton has concentrated on the interaction of inorganic nutrients and carbon in controlling bacterial metabolism, stemming from the assumption that bacterioplankton are carbon limited [14], yet many studies in freshwater indicate that bacterioplankton growth can be limited by nutrients alone rather than in concert with carbon [15]–[17]. Additional studies have consistently shown temporal variability in the elements limiting bacterial metabolism, suggesting that the seasonal dynamics of DOM composition and availability may alter demand for inorganic nutrients [18]–[20]. In addition to affecting the flow of energy and nutrients through the microbial food web, nutrient enrichment may also alter the taxonomic or phenotypic composition of the prokaryotic community, leading to shifts in the utilization of DOM and other resources [21]–[25].

This study sought to clarify the nature of bacterial nutrient limitation in high-elevation lakes of the Sierra Nevada: how nutrient loading directly alters the growth rates, DOM consumption rates, and taxonomic composition of bacterioplankton communities. To test the hypothesis that bacterioplankton in these systems exhibit nutrient limitation independent of indirect phytoplankton responses and in the context of seasonal shifts in DOM source pools, we conducted two distinct *in situ* nitrate- and phosphate-enrichment microcosm experiments in a well-characterized Sierra Nevada lake known to be impacted by ongoing eutrophication [11]. First, we executed a week-long dilution culture experiment following ice-off, when production of autochthonous DOM was at a minimum and organic matter was derived primarily from snowmelt inputs of terrigenous material. While the dark dilution culture approach used in this experiment allowed us quantitative assessment of bacterial community growth rate responses and concomitant consumption of extant DOM in the absence of contemporaneous photosynthetic production, the absence of light and reduced bacterivore density represent an artificially manipulated planktonic community. Our second experiment was designed to examine how unmanipulated bacterial communities responded to nutrients, and was conducted in transparent microcosms during fall overturn when phytoplankton biomass and production were peaking. Community structure shifts in this experiment are more representative of natural conditions, but carbon consumption cannot be measured directly and thus must be inferred by proxy using estimates of bacterial production derived from radiolabeled leucine uptake. The results of these complementary experimental approaches demonstrate clear impacts of phosphate, but not nitrate, on both short-term bacterial growth and community structure independent of phytoplankton nutrient responses, and together provide insight into the nature of phosphorus limitation in oligotrophic waters and the potential responses of bacteria to eutrophication.

## Materials and Methods

### Study site

This research was conducted in Emerald Lake, a cirque lake located on the western slope of the south-central Sierra Nevada (California, USA, 36°35′49″N, 118°40′30″W). Research permits for this study were provided by Sequoia-Kings Canyon National Park. The lake, situated at 2800 m a.s.l. in a sparsely forested granitic catchment, is representative of the >4000 high-elevation lakes (>2500 m a.s.l., >1 ha) scattered throughout the Sierra Nevada in size (2.7 ha, 10.5 m z_max_), solute chemistry, and catchment composition [10]. Three decades of research on the hydrology, chemistry, and biology of Emerald Lake have provided baseline data on ecosystem biogeochemical dynamics, atmospheric deposition, and evidence for continuing eutrophication [11]. Emerald Lake is remote, accessible only by an 8 km hike (with nearly 2 km elevation gain) from the nearest source of potable water and electricity and ∼10 hours travel time from proper laboratory facilities.

### Experimental design

Two separate *in situ* microcosm experiments were conducted in collapsible 10 L low-density polyethylene ‘cubitainers’ (Models 932163 and 250013, Reliance Products, Winnipeg, MB, Canada) which had been leached with 10% HCl for three days, rinsed, dried, then leached again for several hours with water from Emerald Lake just prior to deployment at 2 m depth in the center of the lake suspended on a subsurface-bouyed and anchored thermistor array line. Each experiment consisted of three replicated treatments: treatment (P) was enriched by ∼1.5 µmol L^−1^ K_2_HPO_4_, treatment (N) was enriched by ∼10 µmol L^−1^ KNO_3_, and an unamended Control treatment was enriched with a comparable volume (1‰) of distilled water. These amendment concentrations were selected to double regional maxima of dissolved N and P in accordance with long term trends in Sierra Lakes [11] and to match previous enrichment experiments conducted in the region [26] for the sake of maximizing historical comparability. Ambient concentrations of phosphate are generally below 0.4 µmol L^−1^ in Sierra Nevada Lakes and ambient nitrate concentrations vary widely with snowmelt conditions with seasonal means typically between 3 and 9 µmol L^−1^ [11], [27].

Each experiment used lakewater collected from a discrete 2 m depth interval in the center of the lake with biogeochemical variables and bacterioplankton community structure representative of the entire water column at each time [28]. The first experiment (DARK) was conducted using microcosms constructed of opaque black polyethylene wrapped in mylar to minimize heat absorbance. Lakewater was collected from the center of Emerald Lake at 2 m depth and each of six microcosms (paired replicates assigned to each of three treatments) was promptly filled with 7.5 L of dilution culture: 2.5 L of whole lakewater inoculated into 5 L of 0.2 µm lakewater filtrate which had been gravity-filtered through a nitrocellulose filter to remove all intact cells (GE/Osmonics E02WP14225, 142 mm diameter, 0.22 µm pore-size). Before collecting filtrate, each filter was flushed with 350 mL lakewater to extract filter-derived contaminant organics, and filters were changed after filtering 3 L lakewater to minimize clogging-induced lysis or sorption. Amendments in the DARK experiment increased mean dissolved inorganic nitrogen (DIN) levels from 4.2 µmol L^−1^ to 15.9 µmol L^−1^ and mean soluble reactive phosphate (SRP) levels from 0.04 µmol L^−1^ to 1.6 µmol L^−1^, altering mean starting DIN:total phosphorus (DIN:TP) molar ratios to 2.7, 161, and 59 for P, N, and Control treatments respectively.

The second experiment (LIGHT) was conducted using nine microcosms (three replicates assigned to each of three treatments) constructed of transparent polyethylene filled with 7.5 L whole lakewater collected from the center of Emerald Lake at 2 m depth and promptly amended with nutrients as described above. Nutrient enrichment in the LIGHT experiment increased mean DIN levels from 0.45 µmol L^−1^ to 9.78 µmol L^−1^ and mean SRP levels from 0.04 µmol L^−1^ to 1.8 µmol L^−1^, altering mean starting DIN:TP molar ratios to 0.3, 86, and 5.8 for P, N, and Control treatments respectively. Nutrient amendments in both experiments successfully enriched inorganic nutrient concentrations in treated microcosms above background levels throughout the duration of the experiments (RM-MANOVA between-subjects treatment effect p<0.01). The DIN:TP ratios represent the extremes of the historical range of observed environmental ratios found in Emerald Lake (roughly 0.2 to 200 molar DIN:TP; [11]) and place N and P treatments in the range of respective P and N limitation according to the criteria of Morris and Lewis [29]. The tenfold difference in ambient (Control treatment) nitrate concentration between the two experiments was due to annual snowmelt flushing of nitrate, highlighting the seasonal variability in inorganic N availability in montane lakes [11], [27], [30], [31].

The experiments were conducted between July and September of 2005, a water-year marked by near-record winter snowpack duration. The ice cover on the lake melted in mid-July, such that the summer growing season was relatively short. The DARK dilution culture experiment was executed just after snowmelt, when epilimnetic net primary production was low (4.0 µg C L^−1^ d^−1^) and presumably contributed little to fresh DOM production at that time. The DARK experiment was conducted for one week (27 July through 2 August, 2005) when the lake was characterized by rapid flushing (residence time 12 days), the onset of post-iceoff stratification (T_surf_ 14.2°C, T_9m_ 8.0°C, thermal stability 32 kJ m^-2^), low chlorophyll *a* (Chl a) concentrations (mean epilinion concentrations 0.25 µg L^−1^), and DOM derived largely from terrestrial sources (DOM fluorescence index (FI) of 1.27; Table S1). The LIGHT experiment with an unmanipulated planktonic community was conducted when the lake was well mixed and photosynthetic rates were relatively high (21.5 µg C L^−1^ d^−1^). The LIGHT experiment was conducted for twelve days (18 to 30 September, 2005) when the lake was characterized by longer hydraulic residence time (209 d), post-stratification mixing (T_surf_ 14.9°C, T_9m_ 13.3°C, thermal stability 6 kJ m^−2^), elevated Chl a concentrations (0.74 µg L^−1^), and an increasing contribution of autochthonous DOM sources (FI 1.43; Table S1).

### Sample collection and storage

For each sample (collected every one to three days), microcosms were removed from the lake for two to three hours for processing. Samples collected and parameters measured included the following: particulate carbon, particulate nitrogen, and particulate reactive phosphorus (PC, PN, PRP); total dissolved nitrogen and phosphorus (TDN,TDP); dissolved organic carbon (DOC); dissolved inorganic nitrate + nitrite (DIN); dissolved soluble reactive phosphorus (SRP); prokaryotic abundance; DNA. In addition, LIGHT microcosms were sampled for fluorescent dissolved organic matter (FDOM), Chl *a*, and uptake of ^3^H-leucine. Particulate and dissolved matter were defined operationally as the fractions collected on and passing through a combusted 47 mm Whatman GF/F glass-fiber filter (nominal pore size 0.7 µm), respectively. DOC and FDOM samples were collected in precombusted amber glass EPA vials with teflon septa, acidified and frozen (freeze-thaw tests comparing refrigerated and frozen fluorescent samples from Emerald Lake indicated an average shift of 0.6% in fluorescence intensities and peak ratios, well within the range of instrument variability at these low fluorescence levels). Samples for ^3^H-leucine incorporation consisted of 30 mL whole water collected in acid-washed triple-rinsed polycarbonate centrifuge tubes with polyethylene lids (Product 3118, Nalgene, Rochester, NY) just before departure from the field site and were enriched with ^3^H-leucine immediately upon return to the laboratory, maintaining *in situ* temperatures using insulated pack-coolers throughout travel. Sample collection and handling methods for DNA, prokaryotic abundance, and chlorophyll followed Nelson [28].

### Analytical procedures

The DOM fluorescence index (FI; [32]) was used to determine the relative contribution of allochthonous (terrestrially-derived; low value) and autochthonous (phytoplankton-derived; high value) fulvic acids to the DOM pool of surface waters as has been conducted previously in high-elevation lakes and streams of the Rocky Mountains, USA [33]. The FI was calculated as the ratio of emission intensity at 450 nm to emission intensity at 500 nm under excitation at 370 nm after subtraction of a ddH_2_O blank measured on a Shimadzu RF-1501 spectrofluorometer. Bacterial density was determined by epifluorescence microscopy of 2% formalin-preserved specimens stained with the nucleic-acid fluor DAPI (4′,6′-diamidino-2-phenylindole) at a final concentration of 5 µg mL^−1^ [34]. Abundance was determined as the average of 10 fields (volumes filtered generally yielded 30–300 cells per field). ^3^H-leucine incorporation was measured by incubating 1.7 mL whole water samples in a chilled flowing water bath maintained at *in situ* temperatures with 20 nmol L^−1^ L-[4,5-^3^H] leucine (Amersham TRK636, 63 Ci mmol^−1^) for 90 to 120 minutes and processing according to the microcentrifuge method of Smith and Azam [35]. Concentrations of dissolved and particulate organic and inorganic N and P were measured according to standard methods (see Nelson [28]). Concentrations of DOC were measured according to Carlson et al. [36].

Terminal restriction fragment length polymorphism (TRFLP) of 16S ribosomal subunit DNA genes amplified by the polymerase chain reaction was used as a DNA fingerprinting method for comparison of bacterial community composition [37]. TRFLP amplifcation was done with universal bacterial 16S primers 27F (AGAGTTTGATCMTGGCTCAG, FAM-labeled) and 519R (GWATTACCGCGGCKGCTG, unlabeled) and digested with restriction enzyme HaeIII as previously described [28]. In order to ascribe terminal restriction fragment (TRF) lengths to specific bacterial clades four clone libraries were constructed from environmental DNA samples drawn from the lake [28]. For the present analysis only clones associated with OTUs found in two clone libraries generated within days of the start of each experiment (31 July 2005 and 18 September 2005) were used and singleton clones were excluded (those forming a unique OTU with no other clones >97% sequence identity found in any of the four libraries). We have listed the relevant clones, their taxonomic assignments, and respective GenBank accessions in Table S2, and summarized those data in Table 1. In addition, we built a phylogenetic tree (Fig. S1) of all of the clones listed in Table 1 and Table S2, clearly showing the phylogenetic context of the respective clades and the monophyletic nature of each TRF (a maximum likelihood tree was built using RaxML [38] from sequences aligned to the SILVA 16S database [39] using the SINA alignment utility. Clades were concensus classified to the most reduced taxonomic level (generally Order/Family) where all clone sequences with the same *in silico* TRF length are classified with >95% probability by Bayesian classifier [40] applied to the curated SILVA 16S sequence alignment (v104; [39]). All clones were analyzed by direct amplicon TRFLP to unambiguously assign putative environmental clone taxonomic information directly to peaks in the experimental TRFLP dataset; additional specifics of the TRFLP, cloning, and statistical assignment methods used here may be found in Nelson [28].

**Table 1:** Summary of nutrient-induced shifts in relative abudance of dominant bacterial lineages.

		Represent.	Clone	Amplicon	*In silico*	LIGHT		DARK		Relevant
Phylum	Concensus clade^1^	Accession	count^2^	TRF (bp)^3^	TRF(bp)^3^	N	P	N	P	Figure
Actinobacteria	Microbacteriaceae	EU914094	8	228.23	230		−		−*	5b
Actinobacteria	Actinomycetales	EU914093	18	223.42	227		−*	+*	−	5a,6a
Actinobacteria	Sporichthyaceae	EU914097	20	221.92	225					
Bacteriodetes	Flavobacteriaceae	EU914089	25	518.02	515–517^4^		+*			6d
Bacteriodetes	Chitinophagaceae	EU914013	14	326.16	327		+*			6e
Bacteriodetes	Sphingobacteriales	EU914014	27	31.38	39					
β-proteobacteria	Alcaligenaceae	EU914084	6	216.28	219		+			
β-proteobacteria	Polaromonads	EU914062	9	317.44	319	0	0			5e
β-proteobacteria	Comamonadaceae	EU914088	6	212.21	215				−	5c
β-proteobacteria	Comamonadaceae	EU914006	15	217.90	217		−*			6c
β-proteobacteria	Burkholderiales	EU914083	12	196.28	200	0	0		+*	5d
Cyanobacteria	Subsection I	EU914078	5	220.41	224		−*	0	0	6b
Verrucomicrobia	Opitutaceae	EU914072	7	218.37	222		+			

Symbols are as follows: +  =  increased relative contribution during experiment, −  =  decreased relative contribution during experiment, * = p<0.05, no star  = p<0.1, blank  = p>0.1, 0 =  absent from treatment. Temporal dynamics of treatment responses are detailed in Figs. 5 and 6.

1 Clades are concensus classified to the most reduced taxonomic level where all clone sequences with the same *in silico* TRF length are classified with >95% probability by Bayesian classifier with the curated SILVA 16S sequence alignment. Clades are defined based on monophyletic *in silico* and measured TRF lengths of clones using the tree in Fig. S1.

2 Counts are the number of clones sequenced from Emerald Lake that satisy criteria of both having matching *in silico* TRF lengths across four clone libraries (96 clones each) reported in Nelson (2009) and sharing concensus classification within the SILVA 16S database. Clones and respective classifications are listed in Table S2. Clones are placed in phylogenetic context in a tree in Fig. S1.

3 TRF lengths are from amplicon digestions based on running cloned 16S DNA through TRFLP (left column) and *in silico* digestions based on the location of the restriction site in sequences (right column) of representative clones using the enzyme Hae III. Note that it is common to find discrepancies between predicted and actual TRF lengths; for further details see Nelson (2009).

4 Although the Flavobacteriaceae are split into two distinct clades with monophyletic *in silico* TRF lengths these clades could not be differentiated on actual TRFLP fragment analyses: representative clones from both clades ran at ∼518 bp.

### Data analysis

Repeated measures multivariate analysis of variance (RM-MANOVA, equivalent to “profile analysis”) was used to test the between-subjects null hypothesis of no difference between nutrient-enriched and control treatments across sampling times for a suite of response variables. Replicate microcosms were grouped according to treatment within each timepoint and separate analyses were conducted for each response variable comparing each nutrient with the control. Explicitly, a repeated-measures MANOVA was conducted comparing Control and Treatment replicate groups (separate RM-MANOVA for each N vs. Control and P vs. Control) for each response variable testing the individual effects of time (within-subject effect) and treatment (between-subject effect) and their interaction (time by treatment). The repeated-measures design accounts for within-microcosm variation through time and the MANOVA approach avoids Type I errors caused by violations of the sphericity assumption associated with univariate RM-ANOVA analysis frameworks [41].

TRFLP data from all samples were aligned and analyzed using a novel frequency-based alignment method to avoid the errors associated with integer-based fragment length “binning” [28]. Bacterial community composition data for all samples were collated using relative abundance data (calculated as within-sample relativized eletropherogram peak area for each fragment; [42]) and multivariate community distances were calculated as the Sørenson dissimilarity index [aka Bray-Curtis or Percent Dissimilarity, calculated 1–2W/(A+B) where W is the sum of shared relative abundances and A and B are the sums of relative abundances in individual sample units [43] using PC-ORD software [44]. Nonmetric multidimensional scaling (NMS) was employed to ordinate data in a lower-dimensional space and the multi-response permutation procedure (MRPP) was implemented in PC-ORD to test the null hypothesis of no difference in bacterial community composition between sampling dates and/or treatments.

## Results

### Microbial growth response

**Figure 1:**
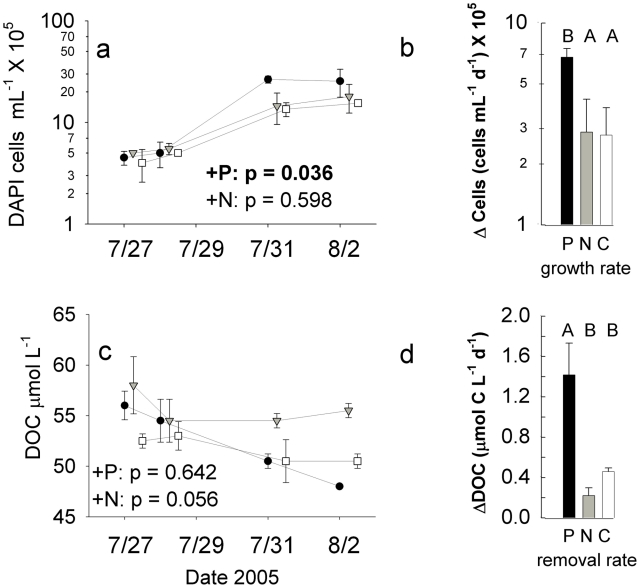
Bacterioplankton abundance and DOC concentration changes over the course of the DARK experiment. Panels (a) and (c) show temporal responses among treatments, and RM-MANOVA p-values are presented for between-subjects treatment effects comparing each nutrient enrichment treatment with the Control, with treatment effects deemed significant and highlighted in **bold** if p<0.05. Letters denote significant differences between treatment means by Tukey-Kramer *post hoc* test at p<0.05. Panels (b) and (d) show rates of change in cell densities and DOC concentrations compared among treatments. Symbols are as follows: •  =  K_2_HPO_4_ additions (+P); ▾  =  KNO_3_ additions (+N), □  =  Control treatments. Error bars are ±1 standard deviation for two replicate microcosms for each treatment. Timepoints are offset for clarity.

In the DARK experiment, P addition increased both the density and growth rates of bacterioplankton above that of both Control and N treatments (Fig. 1a, b). Changes in DOC concentrations were not consistently different between treatments at individual timepoints (RM-MANOVA treatment effect p>0.05; Fig. 1c), likely due to the imprecision in the DOC measurement equaling replicate variance (∼1 µmol L^−1^; [36]), but experiment-wide rates of DOC removal were significantly enhanced in the P treatment relative to the N and Control treatments (p<0.05; Fig. 1d). Moreover, while there was no significant difference in cell concentrations or DOC between treatments at the beginning of the experiment (ANOVA p>0.05) cell abundances were higher and DOC concentrations lower in the P treatment relative to both N and Control treatments at the end of the experiment (ANOVA p<0.05, Tukey post-hoc test P ≠ N = Control at α = 0.05).

**Figure 2:**
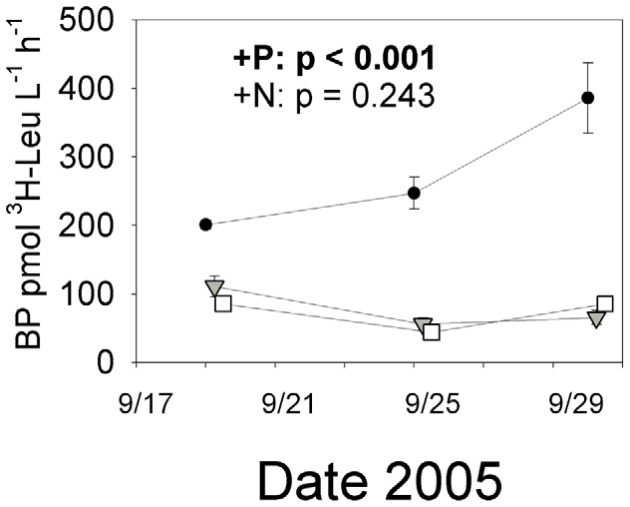
^3^H-leucine incorporation over the course of the LIGHT experiment. Statistics and symbols as for Fig. 1. Within-subjects effects (time and time X treatment) were significant for ^3^H-leucine incorporation (p<0.01). Symbols are as follows: •  =  K_2_HPO_4_ additions (+P); ▾  =  KNO_3_ additions (+N), □  =  Control treatments. Error bars are ±1 standard deviation for three replicate microcosms for each treatment. Timepoints are offset for clarity.

In the LIGHT experiment, leucine incorporation rates in the P treatment increased two- to four-fold above rates in both Control and N treatments and this increase was both rapid and sustained (Fig. 2). The LIGHT experiment exhibited steady increases in FI across all treatments (within-subjects time effect p<0.001, increasing from 1.40 to 1.44 over the course of the experiment), indicating production of autochthonous DOM throughout the experiment but no differential effect of nutrients on autochthonous DOM proportions (RM-MANOVA between-subjects treatment effect p>0.1). There was no concomitant decline in specific UV-A absorbance (commonly associated with allochthonous DOM) in all microcosms (RM-MANOVA within-subjects time effect p>0.1), indicating that the increase in FI was caused by increased production of phytoplankton DOM rather than loss of allochthonous DOM.

### Biogeochemical response

**Figure 3:**
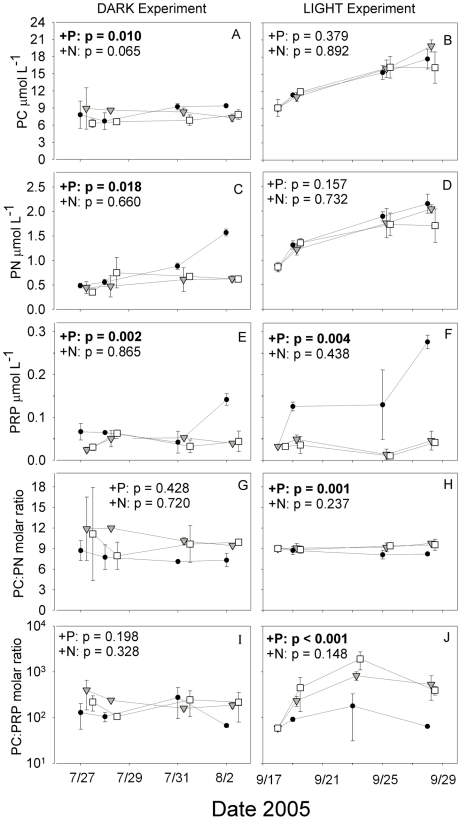
Temporal dynamics of particulate carbon, nitrogen, and phosphorus concentrations and ratios through the two experiments. Symbols are as follows: •  =  K_2_HPO_4_ additions (+P); ▾  =  KNO_3_ additions (+N), □  =  Control treatments. Error bars are ±1 standard deviation for replicate microcosms for each treatment. Timepoints are offset for clarity.

Although all bacterial and biogeochemical variables were measured identically in both experiments (with additional measurements of FDOM, bacterial production, and Chl a appropriate only to the unmanipulated LIGHT experiment) only a subset of particulate organic variables showed significant differential responses (Fig. 3). Phosphorus enrichment increased PC and PN concentrations in the DARK experiment (Figs. 3a and 3c) and PRP concentrations in both experiments (Figs. 3e and 3f), also causing significant declines in PC:PN and PC:PRP in the LIGHT (Figs. 3h and 3j). All microcosms in both experiments exhibited significant decreasing trends in DOC:PC through time (RM-MANOVA within-subjects time effect p<0.01), with this effect driven by DOC drawdown in the DARK experiment (Fig. 1) and PC increase in the LIGHT experiment (Fig. 3b). A significant decline in DIN in the P treatment relative to the control during the LIGHT experiment was observed, along with a similar but nonsignificant decline in DIN in the DARK experiment (RM-MANOVA between-subjects treatment effects p = 0.007 and 0.061, respectively). No significant treatment effect was found through time for dissolved organic N or P in either experiment (RM-MANOVA between-subjects treatment effects all p>0.1). Phytoplankton production of PC and DOM occurred in all microcosms throughout the LIGHT experiment, as evidenced by continuously increasing PC (Fig. 3b) as well as FI and Chl a (data not shown; RM-MANOVA within-subjects time effect p<0.05) across all treatments (RM-MANOVA within-subjects time effect p>0.1). However, there were no differences in rates of change in FI, PC, or Chl a between treatments (RM-MANOVA between-subjects treatment effects all p>0.1), indicating that although primary production was occurring throughout the experiment there was either no significant effect of nutrient addition on algal biomass production or any differential increases in biomass among treatments were removed rapidly by treatment-specific elevated grazing rates.

### Bacterial community response

**Figure 4:**
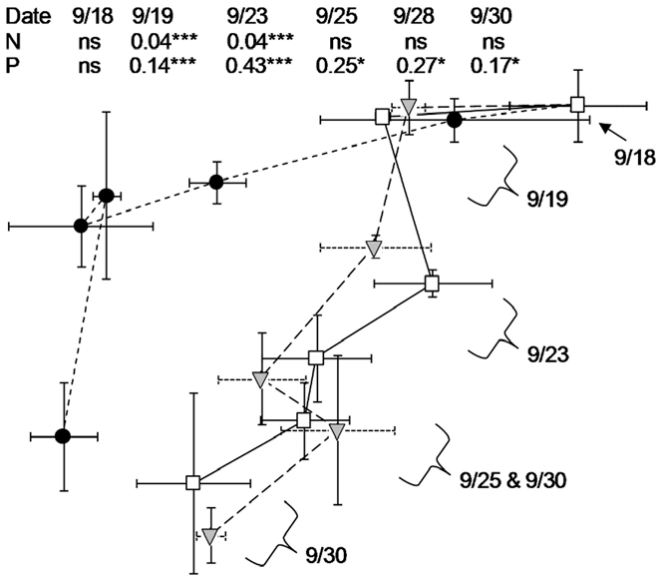
NMS ordination plots of changes in bacterial community composition during the LIGHT experiment. Sampling dates are labeled according to their ordination position using brackets and arrows; lines connecting points follow the temporal trajectory. Results of MRPP comparing significance of each enrichment with the control on each date are presented as tables: values are effect size A, the chance-corrected within-group agreement for each treatment (A = 1 when all members of a group are identical; A<0 indicates more within-group heterogeneity than expected by chance) with significance tests represented as *p<0.05, **p<0.01, ***p<0.001, ns = [A<0 and p>0.05]. The DARK experiment (not shown) exhibited similar differences between P and Control treatments after the third day but no difference between N and Control treatments at any timepoint. Symbols are as follows: •  =  K_2_HPO_4_ additions (+P); ▾  =  KNO_3_ additions (+N), □  =  Control treatments. Error bars are ±1 standard deviation for replicate microcosms for each treatment.

Ordination of TRFLP data via nonmetric multidimensional scaling (NMS) revealed that bacterial community composition shifted through time in both experiments (stress for the two-dimensional NMS solutions were 9.7 for the DARK - not shown - and 11.6 for the LIGHT experiment – Fig. 4). In both experiments P-enrichment produced communities which grew significantly different from both the Control and N-enrichment treatments after just one day and maintained a significantly different community throughout both experiments at each timepoint beyond the initial inoculation (Fig. 4; MRPP was used to statistically contrast mean within-group Sørensen distance with mean between-group Sørensen distance, showing significantly higher between-group difference when comparing P treatments with either N or Control treatments: effect size A>0.14, p<0.001); no concomitant effect was seen for N in either experiment (p>0.05) except for a slight departure from the Control treatment in the LIGHT experiment within the first few days after inoculation (Fig. 4).

At the end of both experiments, but not at the start, community differences between the P and Control treatments were significantly larger than between the N and Control treatments (t-test on mean Sørensen distances between treatments p<0.05). Community structure differences within microcosms between the start and end of the experiment were especially high in the LIGHT experiment (up to 80% difference), indicating a strong temporal shift within the microcosms, but the treatments did not differ significantly in the magnitude of change through time in either experiment (ANOVA p>0.05 in both experiments). There was no significant difference in community composition between treatments at the start of either experiment (MRPP: A<0, p>0.05 in both experiments), nor did treatments differ in the degree of community similarity among replicates at any time (ANOVA p>0.05 in both experiments), demonstrating that filtration and mixing manipulations were applied consistently among treatments and replicates in both experiments. The magnitude of variation between treatments at the start of the experiment was equivalent to the magnitude of variation among replicates (mean Sørensen distances<0.3, all treatments in both experiments), but temporal variation was roughly twice as large (mean Sørensen distances >0.5, all treatments in both experiments), indicating significant community shifts within all microcosms through time. The mean Sørensen distance between P and Control treatments at the end of the experiment was significantly greater than the mean Sørensen distance between N and Control Treatments (t-test p<0.05 in both experiments).

**Figure 5:**
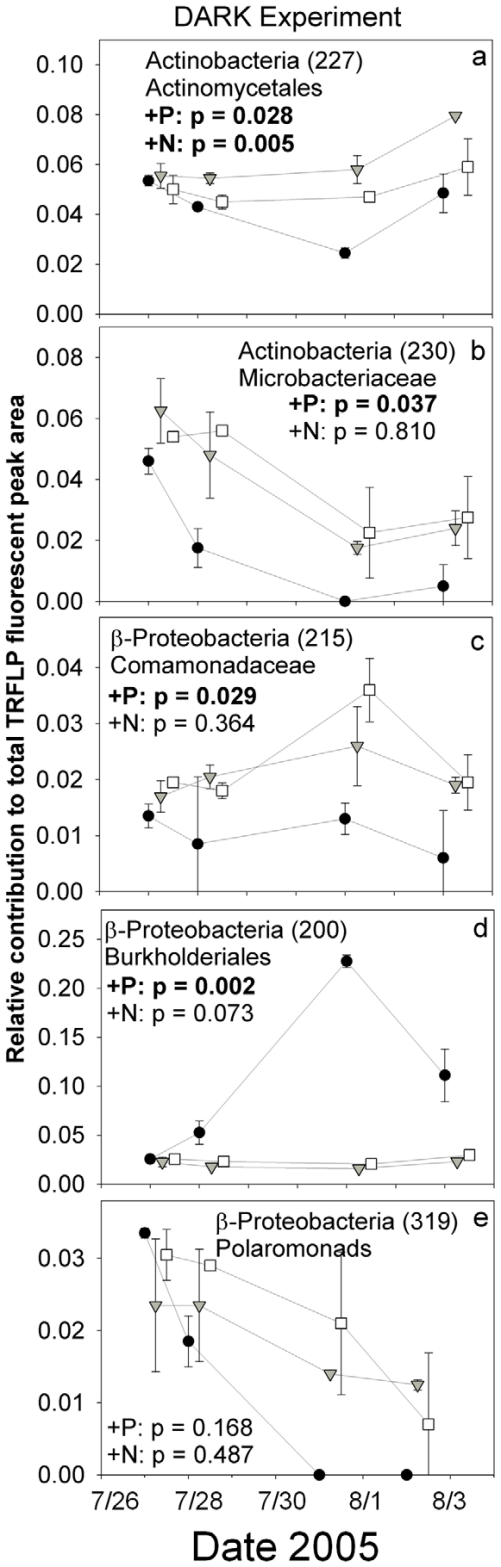
Temporal dynamics of selected TRF relative abundances which exhibited significant treatment effects during the DARK experiment. Note that y-axis values are different for each taxa. Data are relative TRFLP peak fluorescences and should not be interpreted as absolute changes. See Table 1 for details of all TRF responses. Symbols are as follows: •  =  K_2_HPO_4_ additions (+P); ▾  =  KNO_3_ additions (+N), □  =  Control treatments. Error bars are ±1 standard deviation for two replicate microcosms for each treatment. Timepoints are offset for clarity.

**Figure 6:**
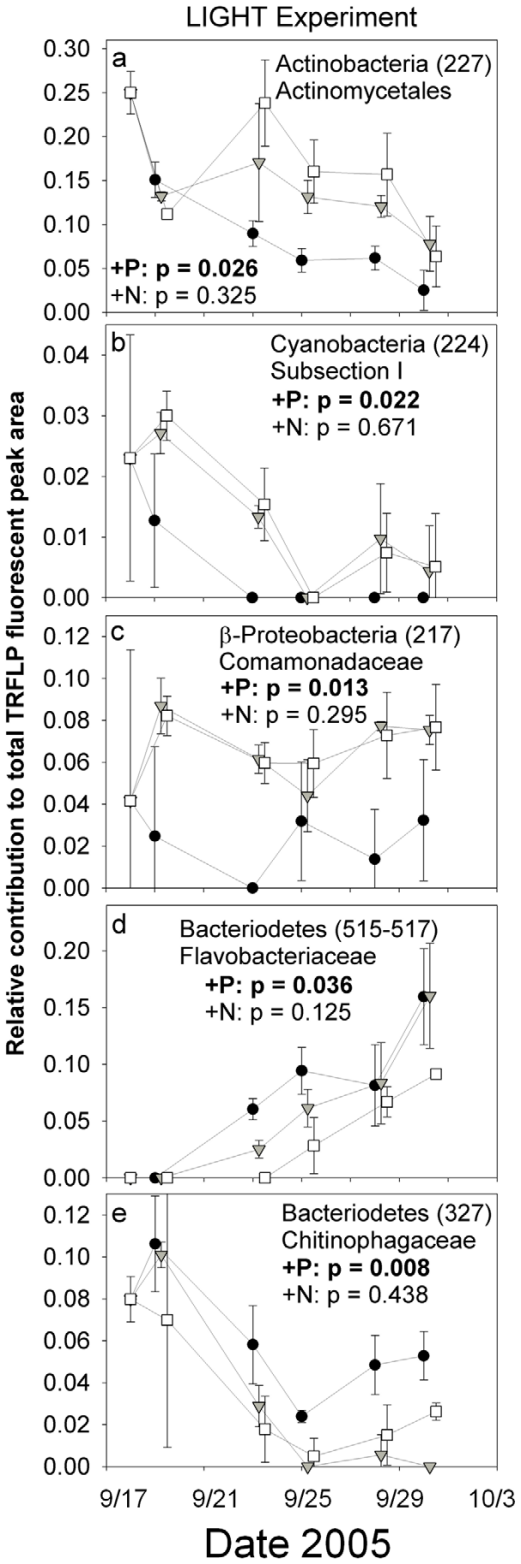
Temporal dynamics of selected TRF relative abundances which exhibited significant treatment effects during the LIGHT experiment. Note that y-axis values are different for each taxa. Data are relative TRFLP peak fluorescences and should not be interpreted as absolute changes. See Table 1 for details of all TRF responses. Symbols are as follows: •  =  K_2_HPO_4_ additions (+P); ▾  =  KNO_3_ additions (+N), □  =  Control treatments. Error bars are ±1 standard deviation for three replicate microcosms for each treatment. Timepoints are offset for clarity.

Responses of specific bacterial taxa to nutrients differed in the two experiments, reflecting the differences in manipulated and unmanipulated communities as well as the difference in available organic and inorganic nutrient pools at the time of the experiments (Table 1). At the broadest taxonomic level, in both experiments P enrichment produced relative declines in two TRFs of Actinomycetes (Table 1, Figs. 5a, 5b, 6a). Two TRFs of Bacteriodetes (Flavobacteriaceae and Chitinophagaceae) increased in the LIGHT experiment in response to P enrichment but showed no clear response in the DARK experiment (Figs. 6d, e). In addition, P enrichment produced a significant decline in the relative abundance of a single Cyanobacteria TRF (belonging to Subsection I) in the LIGHT experiment (Fig. 6b; Cyanobacteria were not detected in the DARK experiment). Responses by the β-proteobacteria to P enrichment were mixed, with one group of Comamonadaceae declining significantly in the LIGHT experiment (Fig. 6c) and two TRFs giving different significant trends in the DARK experiment (Figs. 5c, 5e). There were no significant changes in specific TRFs under N enrichment in the LIGHT treatment, but one Actinomycete TRF, which responded negatively to P, increased significantly in the N-enriched treatment in the DARK experiment as well (Fig. 5a).

## Discussion

In this manuscript, we have quantified in a single ecosystem how a “P pulse” affects bacterial growth (both cell doubling and leucine uptake), organic matter consumption, bacterial growth efficiency, bacterial community structure, and the nutrient stoichiometry of organic matter. We have also contrasted phosphate effects with those of nitrate, another potential agent of eutrophication in these systems. Specifically, our results demonstrate that phosphorus enhances bacterial uptake of extant allochthonous DOM in the dark (Fig. 1), enhances bacterial growth and/or production on both allochthonous (FI 1.27) and autochthonous DOM (FI 1.43) (Figs. 1,2), and causes shifts in the stoichiometry of particulate organic matter (Fig. 3). Our results separate bacterial nutrient responses from from indirect phytoplankton effects, as these patterns were found both in dark dilution cultures and in transparent unmanipulated microcosms when nutrients did not alter phytoplankton biomass, although we cannot rule out differential phytoplankton production in the LIGHT experiment being rapidly removed by grazing pressure. Although the two experiments are not directly comparable because of the differences in nutrient conditions of the ambient environment (Table S1), it is meaningful that in both cases P, not N, engendered shifts in bacterial and biogeochemical variables. Furthermore, our data unambiguously demonstrate that P induces shifts in bacterioplankton community structure rapidly (and similarly in both experiments in the case of the Actinobacteria), and such changes in taxonomic composition may influence the lability of various DOM sources. Rates of DOM removal and particulate organic stoichiometry are very relevant to understanding eutrophication in any habitat, and in particular high-elevation lakes, where eutrophication is typically assessed and defined by chlorophyll or particulate carbon responses in these types of nutrient bioassays [26].

### Bacterioplankton growth responses to nutrient enrichment

The rapid P-enrichment response in leucine uptake exhibited by the microbial community in the LIGHT experiment (Fig. 2) and the significant P-induced increase in both bacterioplankton density and growth rates in the DARK experiment (Fig. 1) are together suggestive of short-term P limitation of bacterioplankton growth in Emerald Lake. These responses occurred both when dissolved organic matter was derived primarily from terrigenous material (DARK experiment, FI 1.27) and when phytoplankton-derived organic matter was produced throughout the experiment (LIGHT experiment, FI 1.43), suggesting that P-induced increases in short term cell growth are independent of organic matter source.

The two experimental approaches provide complementary assessments of microbial growth responses to nutrients. Although the DARK experiment allows simultaneous direct measurement of bacterial growth rates and DOC removal rates (Fig. 1), it requires that microcosms be incubated in the absence of light and with reduced grazer abundance. In contrast the leucine incorporation rates used in the unmanipulated LIGHT experiment are poor proxies for rates of bacterial biomass production: increased leucine uptake may also be attributable to selection of taxa able to utilize leucine as a growth substrate or to a P-induced enhancement of dissimilatory leucine uptake uncoupled from biomass generation and other aspects of bacterial “production.” In addition, the long travel times before the addition of leucine required for this study further reduce our ability to infer realistic growth rates from this proxy measurement.

Previous studies experimentally examining temporal variability in the relationship between nutrient availability and prokaryotic production in high-elevation lakes have revealed similar trends to those observed in this study. Villar-Argaiz, et al. [45] found that P-enrichment of microcosms during fall overturn in a high-elevation lake (La Caldera, Sierra Nevada, España) generated similarly strong responses in bacterial production, and propose that the increases were due to enhanced release of phytoplankton DOM in contrast to an early-season enrichment study under lower-productivity conditions which yielded a subdued bacterial response attributed to limited release of DOM by phytoplankton. Morris and Lewis [18], working in a high-elevation reservoir (Dillon Lake, Rocky Mountains, USA), found significant temporal variability in bacterioplankton nutrient limitation via enrichment of laboratory dilution cultures, but determined that P alone was sufficient to stimulate prokaryotic production in most cases. In the present study, P-enrichment (but not N-enrichment) stimulated prokaryotic growth and caused community shifts in two disparate experiments conducted in different limnological regimes, supporting the conclusions of Morris and Lewis [18] that P-induced stimulation of production and community shifts is a general characteristic of the bacterioplankton communities of Emerald Lake, regardless of seasonal or experimental variations in DOM source.

### Implications for the understanding of Eutrophication and DOM processing

Phosphate enrichment increased the rate of DOC removal during the DARK dilution culture experiment (Fig. 1d). This result suggests that nutrient pulses have the potential to alter short-term ecosystem metabolism by increasing the consumption of DOM by bacterioplankton. Eutrophication is commonly considered a decadal-scale process (e.g. [11]) and it can be argued that short-term nutrient enrichment experiments are uninformative for understanding this process in these systems. However, because alpine lakes are snow-covered for a majority of the year, pulsed nutrient inputs from accumulated atmospheric deposition in snowmelt are a common feature of long-term nutrient pollution, and understanding bacterioplankton responses to such pulses is likely a key component of understanding high-elevation eutrophication.

Because significant DOC removal occurred in the DARK Control treatment, we can infer that the bacterioplankton were not carbon-limited and that P-enrichment only enhanced uptake of an already bioavailable DOM pool. Sadro et al. [46] recently demonstrated that bacterioplankton in Emerald Lake show differential rates of DOC removal on diel scales; we observed P-induced increases in DOC removal when DOM pools were primarily derived from snowmelt runoff rather than phytoplankton production, and further investigation of nutrient-induced DOM removal in this system would benefit greatly from parallel dark dilution studies conducted on different seasonal sources of DOM. A handful of recent studies have investigated the regulation of differential consumption of various (recalcitrant) fractions of DOM in natural environments by P availability [24], [47], [48]. More proximally relevant, a recent experimental study in a similar high-elevation lake found increased productivity and growth efficiency of high-elevation bacterioplankton on phytoplankton-derived DOM relative to soil extracts [49], speculating that the relatively high C:P ratios in the former substrate alleviated nutrient limitation in the bacterioplankton. These results thus illustrate the potential for nutrient enrichment to increase the lability of recalcitrant DOM, particularly terrigenous substrates which are expected to change in the timing and magnitude of inputs with changing regional climate patterns in the Sierra Nevada [50], [51].

Although bacterial growth was enhanced by P amendment in both experiments we cannot say if DOC uptake may have been transferred to higher trophic levels or been respired by bacterioplankton. However, the stoichiometric shifts in particulate nutrient concentrations observed in response to P-enrichment (Fig. 3) together point to increased transfer of organic matter from dissolved to particulate fractions in both experiments. Because P-induced increases in particulate C, N and P (but not carbon:nutrient ratios) were observed in the DARK experiment (Figs. 3a, 3c, 3e), it may be that short-term P amendment enhances either the efficiency of capture of bacterioplankton on GF/F filters (due to size increase or aggregation) or the biomass of microzooplankton grazers. The significant decrease in PC:PRP and PC:PN in P-enriched microcosms during the LIGHT experiment in the absence of P-enhanced primary production (Figs. 3h, 3j) is suggestive of P-induced increases in nutrient uptake by organisms >0.7 µm (including most phytoplankton), but this cannot be directly linked to bacterioplankton growth or size fractionation. The increased rates of nitrate drawdown observed in response to P enrichment in both experiments may be due to stimulation of uptake by heterotrophic bacterioplankton or larger phytoplankton. In this system 16S amplicons with homology to eukaryotic chloroplasts are occasionally amplified in clone libraries but have distinct *in silico* digest lengths, none of which appear to be a major contributer to the patterns of community differentiation shown in Figure 3.

### Bacterial community structure response to nutrient enrichment

The compositional shift in the bacterioplankton community in response to P-enrichment (Fig. 4) was significant and rapid in both experiments, and this contrasted with a lack of overall effect of nitrate amendment on bacterial communities. In addition, the nonsignificant response of FI to nutrient enrichment suggests that the observed modifications to bacterial community structure are driven by P-availability rather than indirect P-induced changes to sources of DOM. P-enrichment appeared to select broadly against Actinobacteria, Cyanobacteria, and some clades of the β-proteobacteria (Comamonadaceae) while selecting for various members of the Bacteriodetes and other clades within the β-proteobacteria (Figs. 5,6). Changes in the relative abundance of specific TRFs must be interpreted with caution, however, as the data derived from TRFLP fingerprinting does not allow for absolute estimations of abundance. As such, a decrease in the relative abundance of a specific taxon may not reflect any significant change in the population size of that taxon, rather the observation may be the result of changes in the abundances of other taxa.

In the present study, Actinobacteria declined in response to P enrichment throughout both experiments (Figs. 5, 6, Table 1). Actinobacteria are relatively well-studied in high-elevation lakes of Europe, where they are primarily derived from autochthonous sources, despite their traditional association with terrestrial habitats [52]. In a recent P-enrichment experiment, Actinobacteria maintained their relative abundance alongside concomitant absolute increases (as measured by actual taxon-specific cell counts) in the abundances of β-proteobacteria and Bacteriodetes [21]. These results suggest that the P-induced declines in various clades of Actinobacteria observed here may be the result of concomitant increases in the population levels of the Bacteriodetes and/or β-proteobacteria (Figs. 5, 6). Further quantitative assessment of clade-specific shifts via fluorescence *in situ* hybridization or quantitiative polymerase chain reaction would help to clarify these responses.

The TRF of Cyanobacteria represented in the LIGHT experiment (Table 1) is represented by a cloned sequence closely related to common oligotrophic marine picocyanobacteria (the top five BlastN hits to the NCBI RefSeq database include 16S genes from the genomes of four *Prochlorococcus* and one *Synechococcus*; e-values<1E−170 with identities >96%); while the depth of our cloning analysis is insufficient to accurately assign a genus-level identification, our observations (Fig. 5b) match with those of Partensky et al. [53] who found that *Prochlorococcus* may be outcompeted under conditions of increased P-availability. There is also some evidence for specialization of various freshwater Bacteriodetes on high-molecular-weight DOM in marine environments and allochthonous material in freshwater habitats, permitting conjecture that they may be a model organism for the many systems displaying P-limited metabolism of recalcitrant DOM [54]. However, the relative abundance of Bacteriodetes was not increased by P-enrichment in the DARK experiment, which would have been expected had P-enrichment increased their ability to consume recalcitrant allochthonous material. Finally, the Verrucomicrobia comprised roughly 6% of the starting community and relative abundances increased with P-enrichment (to ∼10%) during the middle of the LIGHT experiment (significant time X treatment interaction in RM-MANOVA p<0.01; results of univariate ANOVA, p<0.05 on days 266, 268, 271). A similarly rapid initial increase in the relative abundance of the Verrucomicrobia was observed in response to P addition by Lindstrom et al. [23] in microcosms derived from the hypolimnion of temperate, dimictic Lake Siggeforasjön in central Sweden.

### Assignment of phylogenetic identity to TRFLP data

While we have gone to considerable lengths to ensure accurate putative assignment of taxonomic identity to TRFs, we must stress that these assignments are far from definitive. Our method for assigning taxonomic identity is thoroughly detailed above and in a companion study [28] and involves assigning taxonomic identity to environmental clones through alignment to curated taxonomies, calculating *in silico* TRF lengths (Table 1, S2), determining monophyly of *in silico* TRF lengths with maximum likelihood phylogenies (Fig. S1), and measuring representative clone TRF lengths for each OTU to correctly match measured TRF lengths to concensus-classified *in silico* lengths. Although the monophyletic nature of many dominant TRFs found in clone libraries collected at the time of each experiment allowed us to assigned putative phylogenetic identities in this study (Fig. S1), in a broader seasonal study of Emerald Lake many TRFs showed some evidence of polyphyly [28]. Because we cannot definitively rule out polyphyly in this system we have avoided referring to TRF peaks as phylotypes in the present manuscript. Many of the TRF nutrient responses we observed (Figs. 5 and 6) were declines in relative abundance rather than increases, and are unlikely to be caused by enrichment of organisms from different groups exhibiting identical TRFs. However, we hope that by assigning putative taxonomic identities to the TRFs showing nutrient responses in this system we can stimulate hypotheses about the ecology of specific, widespread freshwater bacterioplankton clades.

### Conclusions

The results presented here demonstrate that bacterioplankton growth, DOM removal and community composition respond rapidly to pulse inputs of inorganic nutrients, suggesting that the concept of nutrient limitation by N or P alone may apply to heterotrophic as well as autotrophic microbes. In addition, it is clear from these results that the response of a bacterial community to inorganic nutrients need not be mediated through the response of phytoplankton and subsequent alterations to the pool of dissolved organic matter: here phosphate stimulated bacterioplankton growth on extant terrestrially-derived DOM. The distinct community shifts which emerged under separate experimental conditions as a response to P-enrichment provide a deeper understanding of the factors which structure microbial communities and insight into the nutrient requirements of populations of bacterial taxa in high-elevation lakes.

Emerald Lake is representative of thousands of lakes scattered throughout the the Sierra Nevada, and in many respects is similar to alpine and sub-alpine lakes worldwide. Although limited in spatial and temporal scale, this experiment demonstrates that P enrichment can significantly alter bacterial metabolism and community structure in these environments over short time scales even when the phytoplankton response is muted by the timing of enrichment and nutrient conditions in the ecosystem. Long-term responses of microbial communities to regional nutrient enrichment in these habitats must be examined through monitoring programs and further experiments at broader spatial and temporal scales. Our results do indicate that monitoring programs examining depositional impacts on high-elevation regions may benefit from including studies of bacterial communities, in part because of their complex community composition and key role in organic matter remineralization in these oligotrophic habitats. With our growing understanding of the ways in which the community structure and metabolism of bacterioplankton affect the quantity and composition of dissolved organic matter, we may develop insights into how nutrient enrichment impacts may alter the heterotrophic metabolism of dissolved organic matter across freshwater ecosystem types.

## Supporting Information

Figure S1 **Phylogenetic distributions of clones collected from Emerald Lake labeled according to TRF length and concensus taxonomy.** Note that TRF lengths are highly monophyletic (each TRF matches only one clade). Maximum likelihood dendrogram is built using RaxML from clones aligned to the SILVA curated 16S alignment (v104, Pruesse et al. 2007) using the SINA aligner. Colors overlying clades are matched to specific TRF lengths and named according to concensus classification using Bayesian analysis (Wang et al. 2007) of the SILVA taxonomy. Clones are derived from randomized clone libraries reported in Nelson (2009) and leaves are labeled according to clone name (library_well), representative GenBank Accession (one clone from each 97% sequence identity OTU was accessed), and *in silico* TRF length. All clones are detailed in Table S2.
(PDF)

Table S1 Means and standard deviations of environmental parameters at the start of the DARK and LIGHT experiments and t-test for significant differences between the two dates. Variables exhibiting different mean values between the two dates (p<0.05) are highlighted in bold. For each experiment six samples are averaged, three before the start of the experiment and three from end of the experiment. The three samples are collected from 0.1 m, 2 m, and 4 m depth in the epilimnion of Emerald lake.
(PDF)

Table S2 Clones used to assign monophyletic concensus taxonomies to specific TRFs. Each clone is associated with a representative GenBank Accession number in the same 97% sequence identity cluster according to Nelson (2009). Each clone has an associated in silico TRF length. Representative clones were run through manual TRFLP in duplicate to derive a measured amplicon TRF length. Taxonomic assignments are done by Bayesian analysis of clone alignment within the SILVA reference curated 16S alignment to assign a putative identification. Confidence values are listed in parentheses (blanks<95).
(PDF)
