# Supplementary figures and images for: Differential Response of High-Elevation Planktonic Bacterial Community Structure and Metabolism to Experimental Nutrient Enrichment
Source: PLoS One. 2011 Mar 31;6(3):e18320. doi: 10.1371/journal.pone.0018320 (PMC3069079; doi:10.1371/journal.pone.0018320)

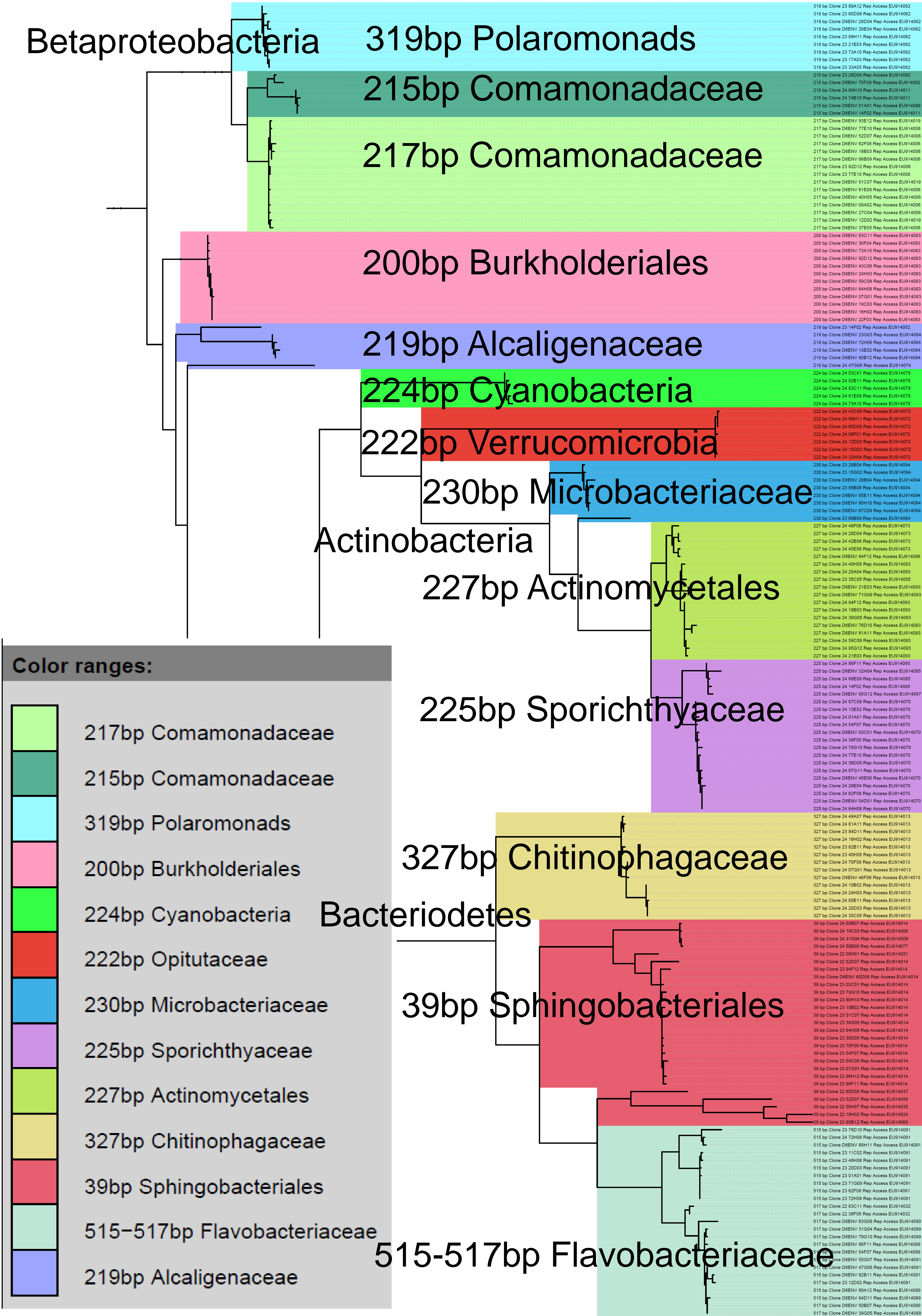

Supplement: Figure S1 — Phylogenetic distributions of clones collected from Emerald Lake labeled according to TRF length and concensus taxonomy. Note that TRF lengths are highly monophyletic (each TRF matches only one clade). Maximum likelihood dendrogram is built using RaxML from clones aligned to the SILVA curated 16S alignment (v104, Pruesse et al. 2007) using the SINA aligner. Colors overlying clades are matched to specific TRF lengths and named according to concensus classification using Bayesian analysis (Wang et al. 2007) of the SILVA taxonomy. Clones are derived from randomized clone libraries reported in Nelson (2009) and leaves are labeled according to clone name (library_well), representative GenBank Accession (one clone from each 97% sequence identity OTU was accessed), and in silico TRF length. All clones are detailed in Table S2. (PDF) [file pone.0018320.s001.pdf]
