# Supplementary material for: Differential Response of High-Elevation Planktonic Bacterial Community Structure and Metabolism to Experimental Nutrient Enrichment
Source: PLoS One. 2011 Mar 31;6(3):e18320. doi: 10.1371/journal.pone.0018320 (PMC3069079; doi:10.1371/journal.pone.0018320)
Supplement: Table S1 — Means and standard deviations of environmental parameters at the start of the DARK and LIGHT experiments and t-test for significant differences between the two dates. Variables exhibiting different mean values between the two dates (p<0.05) are highlighted in bold. For each experiment six samples are averaged, three before the start of the experiment and three from end of the experiment. The three samples are collected from 0.1 m, 2 m, and 4 m depth in the epilimnion of Emerald lake. (PDF) [file pone.0018320.s002.pdf]

Table S1: Means and standard deviations of environmental parameters at the start of the DARK and LIGHT experiments and t-test for significant differences between the two dates.

Variable	Mean DARK	Std Dev DARK	Mean LIGHT	Std Dev LIGHT	t-test Prob > |t|
**Bacterial Density (10^5 cells mL^{-1})**	**9.99**	**2.33**	**21.94**	**7.94**	**0.012**
Bacterial production (pmol ^{3}H Leu L^{-1} h^{-1})	41.9	41.5	33.6	34.2	0.725
**Chl a (ug L^{-1})**	**0.25**	**0.17**	**0.74**	**0.12**	**0.000**
**Nitrate (µmol L^{-1})**	**3.56**	**0.41**	**0.46**	**0.03**	**0.000**
SRP (µmol L^{-1})	0.02	0.02	0.04	0.00	0.193
DOC (µmol L^{-1})	55.6	3.4	60.2	6.2	0.14
DON (µmol L^{-1})	2.88	1.19	2.91	0.58	0.949
**FI**	**1.27**	**0.02**	**1.43**	**0.02**	**0.000**
**PC (µmol L^{-1})**	**9.46**	**1.96**	**12.68**	**2.16**	**0.030**
PN (µmol L^{-1})	1.24	0.29	1.46	0.30	0.249
PRP (µmol L^{-1})	0.03	0.02	0.05	0.02	0.126
**Residence time (days)**	**12**	**7**	**209**	**46**	**0.000**
**Discharge (m^3 d^{-1})**	**19420**	**9665**	**897**	**197**	**0.001**
