# Supplementary material for: Differential Response of High-Elevation Planktonic Bacterial Community Structure and Metabolism to Experimental Nutrient Enrichment
Source: PLoS One. 2011 Mar 31;6(3):e18320. doi: 10.1371/journal.pone.0018320 (PMC3069079; doi:10.1371/journal.pone.0018320)
Supplement: Table S2 — Clones used to assign monophyletic concensus taxonomies to specific TRFs. Each clone is associated with a representative GenBank Accession number in the same 97% sequence identity cluster according to Nelson (2009). Each clone has an associated in silico TRF length. Representative clones were run through manual TRFLP in duplicate to derive a measured amplicon TRF length. Taxonomic assignments are done by Bayesian analysis of clone alignment within the SILVA reference curated 16S alignment to assign a putative identification. Confidence values are listed in parentheses (blanks<95). (PDF) [file pone.0018320.s003.pdf]

| Clone Name (Library_Well) | Representative Genbank Accession (97% Sequence Identity OTU Cluster) | Predicted In Silico HaeIII TRF length | Measured Amplicon HaeIII TRF length | Phylum | Class | Order | Family | Genus |
|---|---|---|---|---|---|---|---|---|
| 24_31G04 | EU914008 | 39 | | Bacteroidetes( | Sphingobacteria(100) | Sphingobacteriales(100) | | |
| 24_19C03 | EU914008 | 39 | | Bacteroidetes( | Sphingobacteria(99) | Sphingobacteriales(99) | Cytophagaceae(96) | |
| D6ENV_60D08 | EU914014 | 39 | 31.38 | Bacteroidetes( | Sphingobacteria(100) | Sphingobacteriales(100) | Cytophagaceae(100) | Arcicella(100) |
| 23_07G01 | EU914014 | 39 | | Bacteroidetes( | Sphingobacteria(100) | Sphingobacteriales(100) | Cytophagaceae(100) | Arcicella(100) |
| 23_10B02 | EU914014 | 39 | | Bacteroidetes( | Sphingobacteria(100) | Sphingobacteriales(100) | Cytophagaceae(100) | Arcicella(100) |
| 23_51C07 | EU914014 | 39 | | Bacteroidetes( | Sphingobacteria(100) | Sphingobacteriales(100) | Cytophagaceae(100) | Arcicella(100) |
| 23_54F07 | EU914014 | 39 | | Bacteroidetes( | Sphingobacteria(100) | Sphingobacteriales(100) | Cytophagaceae(100) | Arcicella(100) |
| 23_94F12 | EU914014 | 39 | | Bacteroidetes( | Sphingobacteria(100) | Sphingobacteriales(100) | Cytophagaceae(100) | Arcicella(100) |
| 23_36D05 | EU914014 | 39 | | Bacteroidetes( | Sphingobacteria(100) | Sphingobacteriales(100) | Cytophagaceae(99) | Arcicella(96) |
| 23_59C08 | EU914014 | 39 | | Bacteroidetes( | Sphingobacteria(100) | Sphingobacteriales(100) | Cytophagaceae(99) | Arcicella(96) |
| 23_03C01 | EU914014 | 39 | | Bacteroidetes( | Sphingobacteria(100) | Sphingobacteriales(100) | Cytophagaceae(97) | Arcicella(97) |
| 23_64H08 | EU914014 | 39 | | Bacteroidetes( | Sphingobacteria(100) | Sphingobacteriales(100) | Cytophagaceae(100) | Arcicella(98) |
| 23_80H10 | EU914014 | 39 | | Bacteroidetes( | Sphingobacteria(100) | Sphingobacteriales(100) | Cytophagaceae(100) | Arcicella(98) |
| 23_86F11 | EU914014 | 39 | | Bacteroidetes( | Sphingobacteria(100) | Sphingobacteriales(100) | Cytophagaceae(100) | Arcicella(98) |
| 23_70F09 | EU914014 | 39 | | Bacteroidetes( | Sphingobacteria(100) | Sphingobacteriales(100) | Cytophagaceae(100) | Arcicella(99) |
| 23_79G10 | EU914014 | 39 | | Bacteroidetes( | Sphingobacteria(100) | Sphingobacteriales(100) | Cytophagaceae(100) | Arcicella(99) |
| 23_96H12 | EU914014 | 39 | | Bacteroidetes( | Sphingobacteria(100) | Sphingobacteriales(100) | Cytophagaceae(99) | Arcicella(99) |
| 22_52D07 | EU914014 | 39 | | Bacteroidetes( | Sphingobacteria(100) | Sphingobacteriales(100) | Cytophagaceae(100) | Arcicella(90) |
| 24_50B07 | EU914014 | 39 | | Bacteroidetes( | Sphingobacteria(100) | Sphingobacteriales(100) | Cytophagaceae(99) | Arcicella(98) |
| 23_39G05 | EU914014 | 39 | | Bacteroidetes( | Sphingobacteria(99) | Sphingobacteriales(99) | Cytophagaceae(98) | Arcicella(96) |
| 22_08H01 | EU914021 | 39 | | Bacteroidetes( | Sphingobacteria(100) | Sphingobacteriales(100) | Cytophagaceae(96) | |
| 22_16H02 | EU914024 | 39 | | Bacteroidetes( | Sphingobacteria(100) | Sphingobacteriales(100) | Sphingobacteriaceae(100) | Mucilaginibacter(100) |
| 22_56H07 | EU914035 | 39 | | Bacteroidetes( | Sphingobacteria(100) | Sphingobacteriales(100) | Sphingobacteriaceae(100) | Mucilaginibacter(100) |
| 22_60D08 | EU914037 | 39 | | Bacteroidetes( | Sphingobacteria(100) | Sphingobacteriales(100) | KD3-93(99) | |
| 23_52D07 | EU914059 | 39 | | Bacteroidetes( | Sphingobacteria(100) | Sphingobacteriales(100) | Sphingobacteriaceae(100) | |
| 23_90B12 | EU914068 | 39 | | Bacteroidetes( | Sphingobacteria(100) | Sphingobacteriales(100) | Sphingobacteriaceae(100) | Mucilaginibacter(100) |
| 24_58B08 | EU914077 | 39 | | Bacteroidetes( | Sphingobacteria(100) | Sphingobacteriales(100) | Cytophagaceae(99) | |
| D6ENV_83C11 | EU914083 | 200 | 196.28 | Proteobacteria | Betaproteobacteria(100) | Burkholderiales(100) | Burkholderiaceae(98) | Polynucleobacter(98) |
| D6ENV_22F03 | EU914083 | 200 | | Proteobacteria | Betaproteobacteria(100) | | | |
| D6ENV_16H02 | EU914083 | 200 | | Proteobacteria | Betaproteobacteria(100) | | | |
| D6ENV_73A10 | EU914083 | 200 | | Proteobacteria | Betaproteobacteria(100) | | | |
| D6ENV_59C08 | EU914083 | 200 | | Proteobacteria | Betaproteobacteria(100) | | | |
| D6ENV_43C06 | EU914083 | 200 | | Proteobacteria | Betaproteobacteria(100) | | | |
| D6ENV_19C03 | EU914083 | 200 | | Proteobacteria | Betaproteobacteria(100) | | | |
| D6ENV_30F04 | EU914083 | 200 | | Proteobacteria | Betaproteobacteria(100) | | | |
| D6ENV_24H03 | EU914083 | 200 | | Proteobacteria | Betaproteobacteria(100) | | | |
| D6ENV_92D12 | EU914083 | 200 | | Proteobacteria | Betaproteobacteria(100) | Burkholderiales(100) | Burkholderiaceae(100) | Polynucleobacter(100) |
| D6ENV_64H08 | EU914083 | 200 | | Proteobacteria | Betaproteobacteria(100) | Burkholderiales(98) | Burkholderiaceae(96) | Polynucleobacter(96) |
| D6ENV_07G01 | EU914083 | 200 | | Proteobacteria | Betaproteobacteria(100) | Burkholderiales(100) | Burkholderiaceae(97) | Polynucleobacter(97) |
| 24_80H10 | EU914011 | 215 | | Proteobacteria | Betaproteobacteria(100) | Burkholderiales(100) | Comamonadaceae(100) | |
| 24_74B10 | EU914011 | 215 | | Proteobacteria | Betaproteobacteria(100) | Burkholderiales(100) | Comamonadaceae(100) | |
| D6ENV_14F02 | EU914011 | 215 | | Proteobacteria | Betaproteobacteria(100) | Burkholderiales(100) | Comamonadaceae(100) | Rhodoferax(100) |
| D6ENV_01A01 | EU914088 | 215 | 212.21 | Proteobacteria | Betaproteobacteria(100) | Burkholderiales(100) | Comamonadaceae(100) | Rhodoferax(99) |
| D6ENV_70F09 | EU914092 | 215 | | Proteobacteria | Betaproteobacteria(100) | Burkholderiales(100) | Comamonadaceae(100) | Rhodoferax(100) |
| 23_28D04 | EU914092 | 215 | | Proteobacteria | Betaproteobacteria(100) | Burkholderiales(100) | Comamonadaceae(100) | |
| D6ENV_52D07 | EU914006 | 217 | 217.90 | Proteobacteria | Betaproteobacteria(100) | Burkholderiales(100) | Comamonadaceae(100) | |

| Clone Name (Library_Well) | Representative Genbank Accession (97% Sequence Identity OTU Cluster) | Predicted In Silico HaeIII TRF length | Measured Amplicon HaeIII TRF length | Phylum | Class | Order | Family | Genus |
|---|---|---|---|---|---|---|---|---|
| D6ENV_09A02 | EU914006 | 217 | | Proteobacteria | Betaproteobacteria(100) | Burkholderiales(100) | Comamonadaceae(100) | |
| D6ENV_37E05 | EU914006 | 217 | | Proteobacteria | Betaproteobacteria(100) | Burkholderiales(100) | Comamonadaceae(100) | |
| D6ENV_66B09 | EU914006 | 217 | | Proteobacteria | Betaproteobacteria(100) | Burkholderiales(100) | Comamonadaceae(100) | |
| D6ENV_27C04 | EU914006 | 217 | | Proteobacteria | Betaproteobacteria(100) | Burkholderiales(100) | Comamonadaceae(100) | |
| D6ENV_77E10 | EU914006 | 217 | | Proteobacteria | Betaproteobacteria(100) | Burkholderiales(100) | Comamonadaceae(100) | |
| D6ENV_18B03 | EU914006 | 217 | | Proteobacteria | Betaproteobacteria(100) | Burkholderiales(100) | Comamonadaceae(100) | |
| D6ENV_62F08 | EU914006 | 217 | | Proteobacteria | Betaproteobacteria(100) | Burkholderiales(100) | Comamonadaceae(100) | |
| 23_77E10 | EU914006 | 217 | | Proteobacteria | Betaproteobacteria(100) | Burkholderiales(100) | Comamonadaceae(100) | |
| 23_92D12 | EU914006 | 217 | | Proteobacteria | Betaproteobacteria(100) | Burkholderiales(100) | Comamonadaceae(100) | |
| D6ENV_40H05 | EU914006 | 217 | | Proteobacteria | Betaproteobacteria(100) | Burkholderiales(100) | Comamonadaceae(100) | Rhodoferax(99) |
| D6ENV_61E08 | EU914006 | 217 | | Proteobacteria | Betaproteobacteria(100) | Burkholderiales(100) | Comamonadaceae(100) | Rhodoferax(99) |
| D6ENV_51C07 | EU914019 | 217 | | Proteobacteria | Betaproteobacteria(100) | Burkholderiales(100) | Comamonadaceae(100) | Rhodoferax(100) |
| D6ENV_12D02 | EU914019 | 217 | | Proteobacteria | Betaproteobacteria(100) | Burkholderiales(100) | Comamonadaceae(100) | Rhodoferax(99) |
| D6ENV_93E12 | EU914019 | 217 | | Proteobacteria | Betaproteobacteria(100) | Burkholderiales(100) | Comamonadaceae(100) | Rhodoferax(99) |
| D6ENV_57A08 | EU914009 | 219 | | Proteobacteria | Betaproteobacteria(100) | Burkholderiales(100) | Alcaligenaceae(100) | GKS98(95) |
| 22_96H12 | EU914026 | 219 | | Proteobacteria | Betaproteobacteria(100) | Burkholderiales(100) | Alcaligenaceae(100) | GKS98(95) |
| 22_70F09 | EU914041 | 219 | | Proteobacteria | Betaproteobacteria(100) | Burkholderiales(100) | Alcaligenaceae(100) | GKS98(95) |
| 23_14F02 | EU914052 | 219 | | Proteobacteria | Betaproteobacteria(99) | | | |
| 24_47G06 | EU914074 | 219 | | Proteobacteria | Betaproteobacteria(97) | | | |
| D6ENV_72H09 | EU914084 | 219 | 216.28 | Proteobacteria | Betaproteobacteria(100) | Burkholderiales(100) | Alcaligenaceae(100) | GKS98(95) |
| D6ENV_23G03 | EU914084 | 219 | | Proteobacteria | Betaproteobacteria(100) | Burkholderiales(100) | Alcaligenaceae(100) | GKS98(100) |
| D6ENV_90B12 | EU914084 | 219 | | Proteobacteria | Betaproteobacteria(100) | Burkholderiales(100) | Alcaligenaceae(100) | GKS98(98) |
| D6ENV_13E02 | EU914084 | 219 | | Proteobacteria | Betaproteobacteria(100) | Burkholderiales(100) | Alcaligenaceae(100) | GKS98(99) |
| 24_15G02 | EU914072 | 222 | 218.37 | Verrucomicrob | Opitutae(100) | Opitutales(100) | Opitutaceae(100) | Opitutus(100) |
| 24_32H04 | EU914072 | 222 | | Verrucomicrob | Opitutae(100) | Opitutales(100) | Opitutaceae(100) | Opitutus(100) |
| 24_06F01 | EU914072 | 222 | | Verrucomicrob | Opitutae(100) | Opitutales(100) | Opitutaceae(100) | Opitutus(100) |
| 24_12D02 | EU914072 | 222 | | Verrucomicrob | Opitutae(100) | Opitutales(100) | Opitutaceae(100) | Opitutus(100) |
| 24_43C06 | EU914072 | 222 | | Verrucomicrob | Opitutae(100) | Opitutales(100) | Opitutaceae(100) | Opitutus(100) |
| 24_60D08 | EU914072 | 222 | | Verrucomicrob | Opitutae(100) | Opitutales(97) | Opitutaceae(97) | Opitutus(97) |
| 24_88H11 | EU914072 | 222 | | Verrucomicrob | Opitutae(100) | Opitutales(99) | Opitutaceae(99) | Opitutus(99) |
| 24_73A10 | EU914078 | 224 | 220.41 | Cyanobacteria | SubsectionI(100) | | | |
| 24_83C11 | EU914078 | 224 | | Cyanobacteria | SubsectionI(100) | | | |
| 24_03C01 | EU914078 | 224 | | Cyanobacteria | SubsectionI(100) | | | |
| 24_61E08 | EU914078 | 224 | | Cyanobacteria | SubsectionI(100) | | | |
| 24_82B11 | EU914078 | 224 | | Cyanobacteria(100) | | | | |
| D6ENV_03C01 | EU914070 | 225 | | Actinobacteria( | Actinobacteria(100) | Actinomycetales(100) | Sporichthyaceae(100) | hgcI_clade(100) |
| 24_13E02 | EU914070 | 225 | | Actinobacteria( | Actinobacteria(100) | Actinomycetales(100) | Sporichthyaceae(100) | hgcI_clade(100) |
| 24_29E04 | EU914070 | 225 | | Actinobacteria( | Actinobacteria(100) | Actinomycetales(100) | Sporichthyaceae(100) | hgcI_clade(100) |
| 24_64H08 | EU914070 | 225 | | Actinobacteria( | Actinobacteria(100) | Actinomycetales(100) | Sporichthyaceae(100) | hgcI_clade(100) |
| 24_67C09 | EU914070 | 225 | | Actinobacteria( | Actinobacteria(100) | Actinomycetales(100) | Sporichthyaceae(100) | hgcI_clade(100) |
| 24_77E10 | EU914070 | 225 | | Actinobacteria( | Actinobacteria(100) | Actinomycetales(100) | Sporichthyaceae(100) | hgcI_clade(100) |
| 24_79G10 | EU914070 | 225 | | Actinobacteria( | Actinobacteria(100) | Actinomycetales(100) | Sporichthyaceae(100) | hgcI_clade(100) |
| 24_87G11 | EU914070 | 225 | | Actinobacteria( | Actinobacteria(100) | Actinomycetales(100) | Sporichthyaceae(100) | hgcI_clade(100) |
| 24_38F05 | EU914070 | 225 | | Actinobacteria( | Actinobacteria(100) | Actinomycetales(100) | | |
| 24_54F07 | EU914070 | 225 | | Actinobacteria( | Actinobacteria(100) | Actinomycetales(100) | | |
| D6ENV_45E06 | EU914070 | 225 | | Actinobacteria( | Actinobacteria(98) | Actinomycetales(98) | | |

| Clone Name (Library_Well) | Representative Genbank Accession (97% Sequence Identity OTU Cluster) | Predicted In Silico HaeIII TRF length | Measured Amplicon HaeIII TRF length | Phylum | Class | Order | Family | Genus |
|---|---|---|---|---|---|---|---|---|
| 24_01A01 | EU914070 | 225 | | Actinobacteria( | Actinobacteria(99) | Actinomycetales(99) | | |
| 24_36D05 | EU914070 | 225 | | Actinobacteria( | Actinobacteria(99) | Actinomycetales(99) | | |
| 24_62F08 | EU914070 | 225 | | Actinobacteria( | Actinobacteria(99) | Actinomycetales(99) | | |
| D6ENV_04D01 | EU914070 | 225 | | Actinobacteria( | Actinobacteria(99) | Actinomycetales(99) | | |
| D6ENV_32H04 | EU914085 | 225 | | Actinobacteria( | Actinobacteria(100) | Actinomycetales(100) | Sporichthyaceae(100) | hgcI_clade(100) |
| 24_14F02 | EU914085 | 225 | | Actinobacteria( | Actinobacteria(100) | Actinomycetales(100) | Sporichthyaceae(100) | hgcI_clade(100) |
| 24_69E09 | EU914085 | 225 | | Actinobacteria( | Actinobacteria(100) | Actinomycetales(100) | Sporichthyaceae(100) | hgcI_clade(100) |
| 24_86F11 | EU914085 | 225 | | Actinobacteria( | Actinobacteria(100) | Actinomycetales(100) | Sporichthyaceae(100) | hgcI_clade(100) |
| D6ENV_95G12 | EU914097 | 225 | 221.92 | Actinobacteria( | Actinobacteria(100) | Actinomycetales(100) | | |
| 23_35C05 | EU914055 | 227 | | Actinobacteria( | Actinobacteria(96) | Actinomycetales(96) | | |
| 24_46F06 | EU914073 | 227 | | Actinobacteria( | Actinobacteria(100) | Actinomycetales(100) | Sporichthyaceae(100) | hgcI_clade(100) |
| 24_45E06 | EU914073 | 227 | | Actinobacteria( | Actinobacteria(100) | Actinomycetales(100) | Sporichthyaceae(100) | hgcI_clade(100) |
| 24_42B06 | EU914073 | 227 | | Actinobacteria( | Actinobacteria(93) | Actinomycetales(90) | | |
| 24_28D04 | EU914073 | 227 | | Actinobacteria( | Actinobacteria(94) | Actinomycetales(90) | | |
| D6ENV_71G09 | EU914093 | 227 | | Actinobacteria( | Actinobacteria(94) | Actinomycetales(94) | | |
| 24_18B03 | EU914093 | 227 | | Actinobacteria( | Actinobacteria(100) | Actinomycetales(100) | Sporichthyaceae(100) | hgcI_clade(100) |
| D6ENV_81A11 | EU914093 | 227 | 223.42 | Actinobacteria( | Actinobacteria(100) | Actinomycetales(100) | Sporichthyaceae(100) | hgcI_clade(100) |
| 24_21E03 | EU914093 | 227 | | Actinobacteria( | Actinobacteria(100) | Actinomycetales(100) | Sporichthyaceae(99) | hgcI_clade(99) |
| 24_25A04 | EU914093 | 227 | | Actinobacteria( | Actinobacteria(100) | Actinomycetales(100) | Sporichthyaceae(99) | hgcI_clade(99) |
| D6ENV_76D10 | EU914093 | 227 | | Actinobacteria( | Actinobacteria(100) | Actinomycetales(100) | Sporichthyaceae(99) | hgcI_clade(99) |
| D6ENV_21E03 | EU914093 | 227 | | Actinobacteria( | Actinobacteria(89) | Actinomycetales(87) | | |
| 24_39G05 | EU914093 | 227 | | Actinobacteria( | Actinobacteria(89) | Actinomycetales(87) | | |
| 24_94F12 | EU914093 | 227 | | Actinobacteria( | Actinobacteria(89) | Actinomycetales(87) | | |
| 24_95G12 | EU914093 | 227 | | Actinobacteria( | Actinobacteria(90) | Actinomycetales(89) | | |
| 24_59C08 | EU914093 | 227 | | Actinobacteria( | Actinobacteria(91) | Actinomycetales(91) | | |
| 24_40H05 | EU914093 | 227 | | Actinobacteria( | Actinobacteria(94) | Actinomycetales(94) | | |
| D6ENV_94F12 | EU914096 | 227 | | Actinobacteria( | Actinobacteria(97) | Actinomycetales(97) | | |
| 23_66B09 | EU914064 | 230 | | Actinobacteria( | Actinobacteria(100) | Actinomycetales(100) | | |
| D6ENV_80H10 | EU914094 | 230 | 228.23 | Actinobacteria( | Actinobacteria(100) | Actinomycetales(100) | Microbacteriaceae(100) | |
| D6ENV_85E11 | EU914094 | 230 | | Actinobacteria( | Actinobacteria(100) | Actinomycetales(100) | Microbacteriaceae(100) | |
| D6ENV_26B04 | EU914094 | 230 | | Actinobacteria( | Actinobacteria(100) | Actinomycetales(100) | Microbacteriaceae(100) | |
| 23_15G02 | EU914094 | 230 | | Actinobacteria( | Actinobacteria(100) | Actinomycetales(100) | Microbacteriaceae(100) | |
| 23_26B04 | EU914094 | 230 | | Actinobacteria( | Actinobacteria(100) | Actinomycetales(100) | Microbacteriaceae(100) | |
| 23_58B08 | EU914094 | 230 | | Actinobacteria( | Actinobacteria(100) | Actinomycetales(100) | Microbacteriaceae(99) | |
| D6ENV_67C09 | EU914094 | 230 | | Actinobacteria( | Actinobacteria(100) | Actinomycetales(100) | Microbacteriaceae(100) | |
| D6ENV_29E04 | EU914062 | 319 | 317.44 | Proteobacteria | Betaproteobacteria(100) | Burkholderiales(100) | Comamonadaceae(100) | Polaromonas(98) |
| 23_60D08 | EU914062 | 319 | | Proteobacteria | Betaproteobacteria(100) | Burkholderiales(98) | Comamonadaceae(95) | |
| 23_88H11 | EU914062 | 319 | | Proteobacteria | Betaproteobacteria(100) | Burkholderiales(100) | Comamonadaceae(98) | |
| 23_73A10 | EU914062 | 319 | | Proteobacteria | Betaproteobacteria(100) | Burkholderiales(100) | Comamonadaceae(99) | |
| 23_21E03 | EU914062 | 319 | | Proteobacteria | Betaproteobacteria(100) | Burkholderiales(98) | | |
| 23_89A12 | EU914062 | 319 | | Proteobacteria | Betaproteobacteria(100) | Burkholderiales(100) | Comamonadaceae(100) | Polaromonas(100) |
| D6ENV_28D04 | EU914062 | 319 | | Proteobacteria | Betaproteobacteria(100) | Burkholderiales(100) | Comamonadaceae(100) | Polaromonas(100) |
| 23_33A05 | EU914062 | 319 | | Proteobacteria | Betaproteobacteria(100) | Burkholderiales(100) | Comamonadaceae(100) | Polaromonas(97) |
| 23_17A03 | EU914062 | 319 | | Proteobacteria | Betaproteobacteria(100) | Burkholderiales(100) | Comamonadaceae(100) | Polaromonas(98) |
| D6ENV_46F06 | EU914013 | 327 | 326.16 | Bacteroidetes( | Sphingobacteria(100) | Sphingobacteriales(100) | Chitinophagaceae(100) | Sediminibacterium(97) |
| 23_82B11 | EU914013 | 327 | | Bacteroidetes( | Sphingobacteria(100) | Sphingobacteriales(100) | Chitinophagaceae(100) | Sediminibacterium(100) |

| Clone Name (Library_Well) | Representative Genbank Accession (97% Sequence Identity OTU Cluster) | Predicted In Silico HaeIII TRF length | Measured Amplicon HaeIII TRF length | Phylum | Class | Order | Family | Genus |
|---|---|---|---|---|---|---|---|---|
| 24_07G01 | EU914013 | 327 | | Bacteroidetes( | Sphingobacteria(100) | Sphingobacteriales(100) | Chitinophagaceae(100) | Sediminibacterium(100) |
| 24_20D03 | EU914013 | 327 | | Bacteroidetes( | Sphingobacteria(100) | Sphingobacteriales(100) | Chitinophagaceae(100) | Sediminibacterium(100) |
| 24_24H03 | EU914013 | 327 | | Bacteroidetes( | Sphingobacteria(100) | Sphingobacteriales(100) | Chitinophagaceae(100) | Sediminibacterium(100) |
| 24_35C05 | EU914013 | 327 | | Bacteroidetes( | Sphingobacteria(100) | Sphingobacteriales(100) | Chitinophagaceae(100) | Sediminibacterium(100) |
| 24_81A11 | EU914013 | 327 | | Bacteroidetes( | Sphingobacteria(100) | Sphingobacteriales(100) | Chitinophagaceae(100) | Sediminibacterium(100) |
| 23_40H05 | EU914013 | 327 | | Bacteroidetes( | Sphingobacteria(100) | Sphingobacteriales(100) | Chitinophagaceae(100) | Sediminibacterium(94) |
| 24_16H02 | EU914013 | 327 | | Bacteroidetes( | Sphingobacteria(100) | Sphingobacteriales(100) | Chitinophagaceae(100) | Sediminibacterium(94) |
| 24_49A07 | EU914013 | 327 | | Bacteroidetes( | Sphingobacteria(100) | Sphingobacteriales(100) | Chitinophagaceae(100) | Sediminibacterium(94) |
| 24_70F09 | EU914013 | 327 | | Bacteroidetes( | Sphingobacteria(100) | Sphingobacteriales(100) | Chitinophagaceae(100) | Sediminibacterium(97) |
| 23_84D11 | EU914013 | 327 | | Bacteroidetes( | Sphingobacteria(100) | Sphingobacteriales(100) | Chitinophagaceae(100) | Sediminibacterium(99) |
| 24_10B02 | EU914013 | 327 | | Bacteroidetes( | Sphingobacteria(100) | Sphingobacteriales(100) | Chitinophagaceae(100) | Sediminibacterium(99) |
| 24_85E11 | EU914013 | 327 | | Bacteroidetes( | Sphingobacteria(100) | Sphingobacteriales(100) | Chitinophagaceae(100) | Sediminibacterium(99) |
| D6ENV_54F07 | EU914089 | 515 | 518.01 | Bacteroidetes( | Flavobacteria(99) | Flavobacteriales(99) | Flavobacteriaceae(99) | |
| D6ENV_89A12 | EU914089 | 515 | | Bacteroidetes( | Flavobacteria(100) | Flavobacteriales(100) | Flavobacteriaceae(100) | |
| D6ENV_84D11 | EU914089 | 515 | | Bacteroidetes( | Flavobacteria(99) | Flavobacteriales(99) | Flavobacteriaceae(99) | |
| D6ENV_88H11 | EU914091 | 515 | | Bacteroidetes( | Flavobacteria(100) | Flavobacteriales(100) | Flavobacteriaceae(100) | Flavobacterium(100) |
| 24_72H09 | EU914091 | 515 | | Bacteroidetes( | Flavobacteria(100) | Flavobacteriales(100) | Flavobacteriaceae(100) | Flavobacterium(99) |
| 23_01A01 | EU914091 | 515 | | Bacteroidetes( | Flavobacteria(100) | Flavobacteriales(100) | Flavobacteriaceae(100) | Flavobacterium(100) |
| 23_48H06 | EU914091 | 515 | | Bacteroidetes( | Flavobacteria(100) | Flavobacteriales(100) | Flavobacteriaceae(100) | Flavobacterium(100) |
| 23_76D10 | EU914091 | 515 | | Bacteroidetes( | Flavobacteria(100) | Flavobacteriales(100) | Flavobacteriaceae(100) | Flavobacterium(100) |
| 23_20D03 | EU914091 | 515 | | Bacteroidetes( | Flavobacteria(100) | Flavobacteriales(100) | Flavobacteriaceae(100) | |
| 23_62F08 | EU914091 | 515 | | Bacteroidetes( | Flavobacteria(98) | Flavobacteriales(98) | Flavobacteriaceae(98) | |
| 23_11C02 | EU914091 | 515 | | Bacteroidetes( | Flavobacteria(98) | Flavobacteriales(98) | Flavobacteriaceae(98) | |
| D6ENV_82B11 | EU914091 | 515 | | Bacteroidetes( | Flavobacteria(99) | Flavobacteriales(99) | Flavobacteriaceae(99) | |
| 23_71G09 | EU914091 | 515 | | Bacteroidetes( | Flavobacteria(99) | Flavobacteriales(99) | Flavobacteriaceae(99) | |
| 23_72H09 | EU914091 | 515 | | Bacteroidetes( | Flavobacteria(99) | Flavobacteriales(99) | Flavobacteriaceae(99) | |
| 22_38F05 | EU914032 | 517 | | Bacteroidetes( | Flavobacteria(100) | Flavobacteriales(100) | Flavobacteriaceae(100) | Flavobacterium(100) |
| 22_83C11 | EU914032 | 517 | | Bacteroidetes( | Flavobacteria(99) | Flavobacteriales(99) | Flavobacteriaceae(99) | |
| D6ENV_79G10 | EU914089 | 517 | 518.02 | Bacteroidetes( | Flavobacteria(100) | Flavobacteriales(100) | Flavobacteriaceae(100) | |
| D6ENV_50B07 | EU914089 | 517 | | Bacteroidetes( | Flavobacteria(100) | Flavobacteriales(100) | Flavobacteriaceae(100) | Flavobacterium(100) |
| D6ENV_86F11 | EU914089 | 517 | | Bacteroidetes( | Flavobacteria(100) | Flavobacteriales(100) | Flavobacteriaceae(100) | Flavobacterium(99) |
| D6ENV_31G04 | EU914089 | 517 | | Bacteroidetes( | Flavobacteria(100) | Flavobacteriales(100) | Flavobacteriaceae(100) | |
| D6ENV_39G05 | EU914089 | 517 | | Bacteroidetes( | Flavobacteria(98) | Flavobacteriales(98) | Flavobacteriaceae(98) | |
| D6ENV_63G08 | EU914090 | 517 | | Bacteroidetes( | Flavobacteria(100) | Flavobacteriales(100) | Flavobacteriaceae(100) | Flavobacterium(98) |
| D6ENV_47G06 | EU914091 | 517 | | Bacteroidetes( | Flavobacteria(100) | Flavobacteriales(100) | Flavobacteriaceae(100) | |
| D6ENV_55G07 | EU914091 | 517 | | Bacteroidetes( | Flavobacteria(98) | Flavobacteriales(98) | Flavobacteriaceae(98) | |
| 23_12D02 | EU914091 | 517 | | Bacteroidetes( | Flavobacteria(99) | Flavobacteriales(99) | Flavobacteriaceae(99) | |
